# ESCMID‐EFISG Survey on Diagnostic and Therapeutic Capacity for Invasive Fungal Infections in Belgium, the Netherlands, and Luxembourg: A Focus on High Azole Resistance

**DOI:** 10.1111/myc.70092

**Published:** 2025-07-12

**Authors:** Robina Aerts, Lize Cuypers, Eelco F. J. Meijer, Michel Kohnen, Jacques F. Meis, Oliver A. Cornely, Katrien Lagrou, Jon Salmanton‐García, Alex Wagemakers, Stefan Vestjens, Peter Verbeeck, Geert Vanheule, Marc Vandevelde, Jens van Van Praet, Bruno van Van Herendael, Jaap van Doesum, Karin van Van Dijk, Alieke van der Van der Hoeven, Wendy W. J. van de Van de Sande, Marijke Reynders, Ann Packeu, Yarah Overmeire, Claudy Oliveira‐dos‐Santos, Eric Nulens, Alexandre Mzabi, Astrid Muyldermans, Isabel Montesinos, Willem Melchers, Johan Maertens, Karel Maelegheer, Sybren Lekkerkerk, Nathalie Layios, Eva Kolwijck, Stijn Jonckheere, Louis Ide, Marie‐Pierre Hayette, Ferry Hagen, Pieter‐Jan Haas, Truus Goegebuer, Peggy Godschalk, Anne‐Isabelle de Moreau, Emmanuel de Laere, Julien de Greef, Deborah de Geyter, Peter Croughs, Philippe Clevenbergh, Jolien Claessens, Ga‐Lai Chong, Reinoud Cartuyvels, Jochem Buil, Roger Bruggemann, Jerina Boelens, An Boel, Robbert Bentvelsen

**Affiliations:** ^1^ Department of Haematology Leuven University Hospitals Leuven Belgium; ^2^ Department of Microbiology Immunology and Transplantation, KU Leuven Leuven Belgium; ^3^ KU Leuven, Department of Microbiology, Immunology and Transplantation, Laboratory of Clinical Microbiology Leuven Belgium; ^4^ Department of Laboratory Medicine, National Reference Centre for Rotavirus University Hospitals of Leuven Leuven Belgium; ^5^ Department of Medical Microbiology and Immunology Canisius‐Wilhelmina Hospital (CWZ)/Dicoon Nijmegen the Netherlands; ^6^ Radboudumc‐CWZ Center of Expertise for Mycology Nijmegen the Netherlands; ^7^ National Service of Infectious Diseases, Centre Hospitalier de Luxembourg Luxembourg Luxembourg; ^8^ Institute of Translational Research, Cologne Excellence Cluster on Cellular Stress Responses in Aging‐Associated Diseases (CECAD), Faculty of Medicine and University Hospital Cologne University of Cologne Cologne Germany; ^9^ Department I of Internal Medicine, Center for Integrated Oncology Aachen Bonn Cologne Duesseldorf (CIO ABCD) University of Cologne Cologne Germany; ^10^ Department I of Internal Medicine, European Confederation for Medical Mycology (ECMM) Excellence Center University of Cologne Cologne Germany; ^11^ German Centre for Infection Research (DZIF), Partner Site Bonn‐Cologne Cologne Germany; ^12^ Clinical Trials Centre Cologne (ZKS Köln), Faculty of Medicine, University Hospital Cologne University of Cologne Cologne Germany; ^13^ Department of Laboratory Medicine University Hospitals Leuven Leuven Belgium; ^14^ National Reference Center for Mycosis University Hospitals Leuven Leuven Belgium

**Keywords:** antifungal resistance, aspergillus antigen, azole‐resistance, invasive aspergillosis, invasive fungal infections, lateral flow assays, lateral flow devices, mycology

## Abstract

**Introduction:**

Invasive fungal infections (IFI) are a major clinical challenge, particularly in immunocompromised patients, and are associated with high morbidity and mortality. With the increasing prevalence of immunosuppressive conditions and ageing populations, the incidence of IFI is rising globally.

**Objective:**

This survey aims to evaluate the diagnostic and therapeutic capacities for IFI in Belgium, the Netherlands, and Luxembourg (Benelux), a region of high azole‐resistance among *Aspergillus fumigatus* isolates.

**Methods:**

A survey evaluating the diagnostic and therapeutic capacity for IFI was conducted in the Benelux. Data were collected from specialists via an online case report form between March and September 2023. The survey addressed patient characteristics, access to microbiology labs, diagnostic methods (microscopy, culture, molecular diagnostics, etc.), IFI incidence, and the availability of antifungal drugs and therapeutic drug monitoring.

**Results:**

In total, 32 hospitals responded to the questionnaire (12 [38%] from the Netherlands, 19 [59%] from Belgium and one [3%] from Luxembourg). Antifungal susceptibility tests were available in 29 institutions (91%), constituting 84% of the centres in Belgium and 100% for the Netherlands (*p* = 0.265). *Aspergillus* PCR testing was available in 12 centres in Belgium (63%) while in 11 centres in the Netherlands (92%, *p* = 0.108). Mucorales PCR testing was available in 56% of centres. Treatment with at least one amphotericin B formulation was only available in 84% of the responding centres. Therapeutic drug monitoring (TDM), although recommended, was possible for voriconazole in 26 centres (81%) while for posaconazole in 24 centres (75%). Significantly more testing (diagnostic tests and TDM) was outsourced in Belgium compared to the Netherlands (*p* < 0.001).

**Conclusions:**

Antifungal susceptibility testing is widely available in Belgium and the Netherlands, but implementation in areas with high azole resistance for *Aspergillus fumigatus* is not yet universal, and techniques vary. Tests for coinfections, like Mucorales PCR, were only available in half of the centres. More testing is outsourced in Belgium, likely due to differences in reference centre organisation, country size, transport, and reimbursement. Delays in diagnosis can impact patient outcomes, so awareness of test availability and transport times is crucial.

## 1 Introduction

Invasive fungal infections (IFI) pose significant challenges in clinical management due to their elusive nature and potentially fatal outcomes, particularly in immunocompromised patients. The increasing prevalence of immunosuppressive conditions, the introduction of new immunomodulatory therapies, and the aging of the population have contributed to the rising incidence of IFI worldwide [1, 2]. Approximately 254,491 patients in the Netherlands (1.5% of the population) and 233,000 (2.1%) in Belgium are estimated to suffer from any fungal infection annually [3, 4]. Prompt and accurate diagnosis followed by appropriate antifungal therapy, in a multidisciplinary setting, is crucial for improving patient outcome [5, 6, 7]. The diagnosis of IFI is highly determined by the repertoire of available diagnostic tests. In certain contexts, pre‐emptive or diagnostic‐driven treatment strategies are being recommended, aligning with principles of antifungal stewardship. But the choice of the diagnostic approach for IFI should be individualised to each centre in line as much as possible with the existing consensual global guidelines, taking into account the local epidemiology of IFI and, as important, the availability of diagnostic tests [5, 7, 8, 9, 10, 11].

Recently, a large survey evaluating the diagnostic and therapeutic capacity for the management of IFI in Europe was performed [12]. It showed that the diagnostic armamentarium was dependent on the gross domestic product (GDP) of the countries. However, no results from Luxembourg were available, and since the countries were grouped by GDP, it was not possible to evaluate results on a country level. Therefore, it was estimated that a local spin‐off in Belgium, the Netherlands, and Luxembourg (Benelux) could provide additional insights. In addition, with the incidence of azole‐resistant *Aspergillus fumigatus* being rather high in Belgium and especially in the Netherlands, zooming in on diagnostics contributing to the detection of resistance and resistance mechanisms in this part of Europe is of importance. *Aspergillus fumigatus* azole‐resistance was first reported from the Netherlands in the late 1990s [13, 14, 15], few years after the introduction of itraconazole in 1990, and has since seen a steady increase both in clinical and environmental isolates challenging the clinical management of aspergillosis [16]. The Benelux is a densely populated region, making up 5%–6% of the European population. GDP in the Benelux is high (> €40,000/resident) according to the Organisation for Economic Co‐operation and Development [17]. *Aspergillus fumigatus* azole‐resistance in Belgium [18, 19] and the Netherlands [20, 21, 22, 23, 24, 25] lies between 7% and 13%. Candidiasis and aspergillosis are the most frequent IFI [3, 4, 24].

This manuscript presents a comprehensive survey of diagnostic modalities for IFI, encompassing traditional microbiological techniques, molecular assays, and logistic considerations. Furthermore, we discuss the availability and accessibility of antifungal agents, considering factors such as prescription guidelines, reimbursement policies, and regional variations in healthcare infrastructure. By synthesising the available diagnostic tests and antifungal treatments, we aim to provide clinicians and policymakers with insights into the diagnostic landscape and therapeutic options in this region of high *Aspergillus fumigatus* azole‐resistance, facilitating informed decision making and optimising patient care strategies.

## 2 Methods

A survey evaluating the diagnostic and therapeutic capacity for IFI was performed in the Benelux. Data were collected via an online electronic case report form hosted at www.clinicalsurveys.net/uc/IFI_management_capacity/ (EFS Summer 2021, TIVIAN GmbH, Cologne, Germany) between March and September 2023. The survey was sent to a selection of Belgian hospital laboratories of interest. In the Netherlands, the survey was spread by the general newsletter from the NVMM (Dutch Society for Medical Microbiology by its Dutch abbreviation Nederlandse Vereniging voor Medische Microbiologie) with a reminder to a selection of Dutch hospitals of interest for which responses were missing. In Luxembourg, the survey was spread via professional contacts. The survey includes patient characteristics, access to microbiology laboratories, mycological diagnostic procedures performed (including microscopy, culture, serology, antigen detection, molecular diagnostics, and antifungal susceptibility testing), IFI incidence of varying pathogens, as well as the availability of antifungal drugs and their therapeutic drug monitoring. A selection of variables can be found in Table 1. The full survey can be found in the Data S1. Fisher's Exact Test was used to determine whether there were significant differences between Belgian and Dutch responses. Luxembourg was not included in this comparison. Statistical analyses were conducted using the statistical software Rstudio (version 2023.12.1 + 402).

**TABLE 1:** Availability of diagnostic possibilities.

	Overall		BE		NL		Comparing BEL vs. NL	LU	
	*n*	%	*n*	%	*n*	%	*p*	*n*	%
Microscopy	29	90.6	18	94.7	11	91.7		1	100.0
Methodologies									
Calcofluor white	22	68.8	14	73.7	8	66.7	0.704	0	0.0
Giemsa stain	17	53.1	9	47.4	8	66.7	0.461	0	0.0
China/India ink	20	62.5	10	52.6	10	83.3	0.128	0	0.0
Potassium hydroxide	17	53.1	10	52.6	7	58.3	1.000	0	0.0
Silver stain	10	31.3	4	21.1	6	50.0	0.127	0	0.0
Microscopy frequency if IFI suspicion									
Never	2	6.3	1	5.3	1	8.3		0	0.0
Rarely	1	3.1	1	5.3	0	0.0		0	0.0
Sometimes	2	6.3	2	10.5	0	0.0		0	0.0
Often	5	15.6	3	15.8	2	16.7		0	0.0
Always	22	68.8	12	63.2	9	75.0	0.697	1	100.0
Access to fluorescence dye	25	78.1	15	78.9	10	83.3	1.000	0	0.0
Direct examination in body fluids when cryptococcosis suspicion									
India ink	20	62.5	10	52.6	10	83.3	0.128	0	0.0
Other dyes	5	15.6	4	21.1	1	8.3	0.624	0	0.0
Silver stain if pneumocystis suspicion	8	25.0	3	15.8	5	41.7	0.206	0	0.0
Direct microscopy if mucormycosis suspicion	17	53.1	9	47.4	8	66.7	0.461	0	0.0
Culture and fungal identification	32	100.0	19	100.0	12	100.0	1.000	1	100.0
Blood cultures when fungemia suspicion?	28	87.5	16	84.2	11	91.7	1.000	1	100.0
Fungal culture methods									
Agar Niger	4	12.5	2	10.5	2	16.7	0.630	0	0.0
Chromogen	18	56.3	10	52.6	8	66.7	0.484	0	0.0
Lactrimel Agar	2	6.3	0	0.0	2	16.7	0.142	0	0.0
Potato Dextrose Agar	10	31.3	3	15.8	7	58.3	***0.021***	0	0.0
Sabouraud dextrose agar	29	90.6	16	84.2	12	100.0	0.265	1	100.0
Sabouraud dextrose agar + Chloramphenicol	23	71.9	14	73.7	9	75.0	1.000	0	0.0
Sabouraud dextrose agar + Gentamicin	18	56.3	10	52.6	8	66.7	0.484	0	0.0
Available tests for specific identification	32	100.0	19	100.0	12	100.0	1.000	1	100.0
Automated identification (i.e., VITEK. other commercial tests)	16	50.0	8	42.1	8	66.7	0.273	0	0.0
Biochemical tests (classic mycology)	17	53.1	11	57.9	6	50.0	0.724	0	0.0
DNA sequencing	17	53.1	7	36.8	10	83.3	***0.024***	0	0.0
MALDI‐TOF MS	30	93.8	17	89.5	12	100.0	0.510	1	100.0
Mounting medium	9	28.1	4	21.1	5	41.7	0.253	0	0.0
Antifungal susceptibility tests?	29	90.6	16	84.2	12	100.0	0.265	1	100.0
Only for yeasts	13	40.6	9	47.4	3	25.0		1	100.0
For both	16	50.0	7	36.8	9	75.0		0	0.0
Antifungal susceptibility technology									
Broth microdilution – CLSI standards	12	37.5	8	42.1	4	33.3	0.717	0	0.0
Broth microdilution – EUCAST standards	15	46.9	7	36.8	7	58.3	0.288	1	100.0
E‐test	12	37.5	7	36.8	5	41.7	1000	0	0.0
VITEK	13	40.6	5	26.3	8	66.7	*0.060*	0	0.0
Serology	26	81.3	14	73.7	11	91.7	0.363	1	100.0
Aspergillus spp.	25	78.1	13	68.4	11	91.7	0.202	1	100.0
Onsite	13	40.6	4	21.1	9	75.0		0	0.0
Outsourced	12	37.5	9	47.4	2	16.7		1	100.0
Candida spp.	17	53.1	10	52.6	7	58.3	1.000	0	0.0
Onsite	5	15.6	2	10.5	3	25.0		0	0.0
Outsourced	12	37.5	8	42.1	4	33.3		0	0.0
Histoplasma spp.	21	65.6	13	68.4	8	66.7	1.000	0	0.0
Onsite	3	9.4	0	0.0	3	25.0		0	0.0
Outsourced	18	56.3	13	68.4	5	41.7		0	0.0
Antigen detection	31	96.9	18	94.7	12	100.0	1.000	1	100.0
Aspergillus, spp. any	30	93.8	18	94.7	11	91.7	1.000	1	100.0
Aspergillus LFT	24	75.0	13	68.4	11	91.7	0.202	0	0.0
Onsite	13	40.6	3	15.8	10	83.3		0	0.0
Outsourced	11	34.4	10	52.6	1	8.3		0	0.0
Aspergillus GM ELISA	29	90.6	18	94.7	10	83.3	0.544	1	100.0
Onsite	19	59.4	10	52.6	8	66.7		1	100.0
Outsourced	10	31.3	8	42.1	2	16.7		0	0.0
Candida antigen	11	34.4	6	31.6	5	41.7	0.705	0	0.0
Onsite	5	15.6	2	10.5	3	25.0		0	0.0
Outsourced	6	18.8	4	21.1	2	16.7		0	0.0
Cryptococcus spp., any	27	84.4	16	84.2	10	83.3	1.000	1	100.0
Cryptococcus LFT	20	62.5	10	52.6	9	75.0	0.274	1	100.0
Onsite	12	37.5	3	15.8	8	66.7		1	100.0
Outsourced	8	25.0	7	36.8	1	8.3		0	0.0
Cryptococcus LAT	18	56.3	14	73.7	4	33.3	*0.060*	0	0.0
Onsite	8	25.0	5	26.3	3	25.0		0	0.0
Outsourced	10	31.3	9	47.4	1	8.3		0	0.0
Histoplasma spp.	17	53.1	10	52.6	7	58.3	1.000	0	0.0
Onsite	2	6.3	1	5.3	1	8.3		0	0.0
Outsourced	15	46.9	9	47.4	6	50.0		0	0.0
Beta‐glucan	25	78.1	15	78.9	9	75.0	1.000	1	100.0
Onsite	5	15.6	2	10.5	3	25.0		0	0.0
Outsourced	20	62.5	13	68.4	6	50.0		1	100.0
Molecular tests	32	100.0	19	100.0	12	100.0	1.000	1	100.0
Aspergillus PCR	24	75.0	12	63.2	11	91.7	0.108	1	100.0
Onsite	14	43.8	5	26.3	9	75.0		0	0.0
Outsourced	10	31.3	7	36.8	2	16.7		1	100.0
Candida PCR	18	56.3	9	47.4	9	75.0	0.158	0	0.0
Onsite	9	28.1	2	10.5	7	58.3		0	0.0
Outsourced	9	28.1	7	36.8	2	16.7		0	0.0
Pneumocystis PCR	31	96.9	18	94.7	12	100.0	1.000	1	100.0
Onsite	21	65.6	9	47.4	12	100.0		0	0.0
Outsourced	10	31.3	9	47.4	0	0.0		1	100.0
Mucorales PCR	18	56.3	10	52.6	7	58.3	1.000	1	100.0
Onsite	6	18.8	2	10.5	4	33.3		0	0.0
Outsourced	12	37.5	8	42.1	3	25.0		1	100.0

*Note:* *p*‐value below 0.05 in bold/italic, trend to significance in italic.

Abbreviations: BE, Belgium; CLSI, Clinical and Laboratory Standards Institute; DNA, deoxyribonucleic acid; ELISA, enzyme‐linked immunosorbent assay; EUCAST, European Committee on Antimicrobial Susceptibility Testing; GM, galactomannan; IFI, invasive fungal infection; LAT, latex agglutination test; LFT, lateral flow test; LU, Luxembourg; MALDI‐TOF MS, matrix‐assisted laser desorption/ionisation time‐of‐flight mass spectrometry; NL, Netherlands; PCR, polymerase chain reaction.

## 3 Results

**FIGURE 1:**
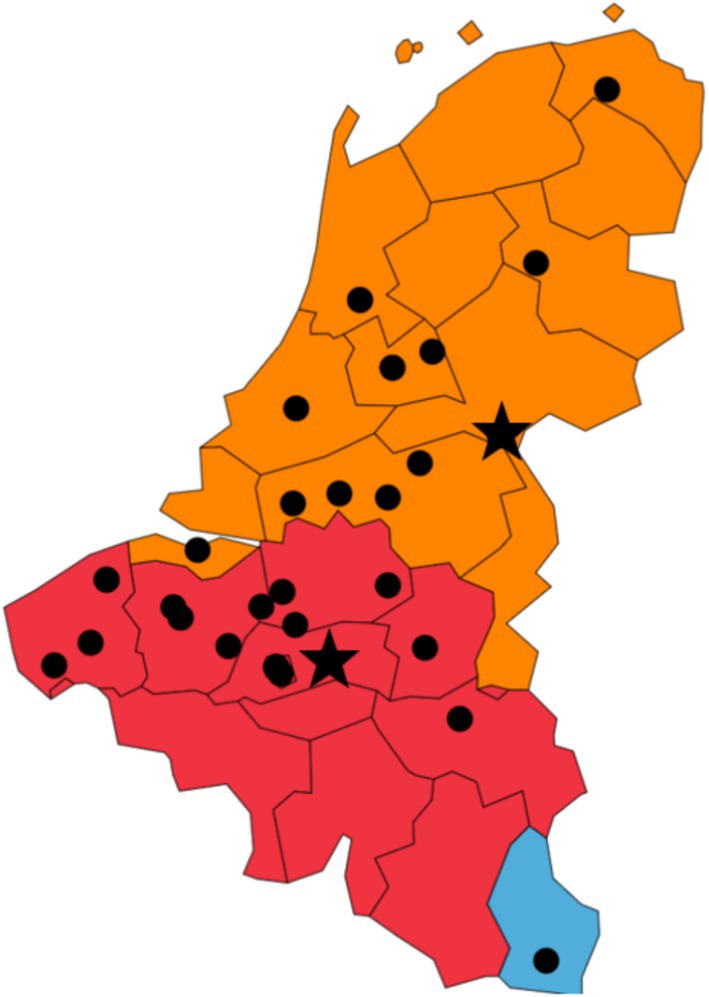
Geographic distribution of the centres that have participated in the survey. Belgium (red, *n* = 19 centres; OLV Ziekenhuis in Aalst, AZ Rivierenland in Bornem, AZ Sint‐Jan and AZ Sint‐Lucas in Bruges, Sciensano, Brussels University Hospital, and CHU Brugmann in Brussels, Labo Nuytinck in Evergem, Ghent University Hospital and Az Jan Palfijn in Ghent, Jessa Ziekenhuis in Hasselt, Jan Yperman Hospital in Ieper, Hôpitaux IRIS SUD in Ixelles, UZ Leuven in Leuven, CHU de Liège in Liège, AZ Sint‐Maarten in Mechelen, H. Hartziekenhuis Mol in Mol, AZ Delta in Roeselare, and GZA Ziekenhuizen Antwerp in Wilrijk). Netherlands (orange, *n* = 12 centres; Meander Medisch Centrum in Amersfoort, AmsterdamUMC and Antoni van Leeuwenhoek in Amsterdam, University Medical Center Groningen in Groningen, Canisius‐Wilhelmina Ziekenhuis and Radboud University Medical Center in Nijmegen, ErasmusMC in Rotterdam, Jeroen Bosch Ziekenhuis in s‐Hertogenbosch, Microvida Laboratory for Medical Microbiology and Immunology across Tilburg, Breda, Roosendaal, and Terneuzen, University Medical Center Utrecht and Westerdijk Fungal Biodiversity Institute in Utrecht, and Isala Hospitals in Zwolle). Luxembourg (blue, *n* = 1 centre; Centre Hospitalier de Luxembourg in Luxembourg). Starts indicate European Confederation of Medical Mycology (ECMM) Excellence Centres in Leuven, Belgium and Nijmegen, Netherlands (https://www.ecmm.info/ecmm‐excellence‐centers/excellence‐centers/).

In total, 32 hospital laboratories (12 [37.5%] from the Netherlands, 19 [59.4%] from Belgium and one [3.1%] from Luxembourg) have responded to the questionnaire (Figure 1). Of the 32 responding hospitals, eight (25.0%) reported a very low incidence of IFI, 11 (34.4%) a low incidence, seven (21.9%) a moderate incidence, four (12.5%) a high incidence and two (6.3%) a very high incidence. The most important pathogens were *Candida* spp. (first place, 93.8%) and *Aspergillus* spp. (second place, 90.6%), followed by *Cryptococcus* spp., Mucorales, *Fusarium* spp. and *Histoplasma* spp. in the third to sixth places respectively (Table 1).

Availability of diagnostic possibilities is summarised in Table 1. Mycological diagnostic procedures were performed exclusively onsite in 17 (53.1%) centres, partially outsourced in 13 (40.6%) centres and completely outsourced in one centre (3.1%). In 22 out of 32 (68.6%) centres reported, microscopy was always performed when IFI is suspected (63.2% in Belgium vs. 75.0% in the Netherlands, *p* = 0.697). Microscopy was only available in 90.6% as also two public health centres not receiving clinical patient samples participated in the survey. Direct examination was performed in case of suspicion of mucormycosis in 17 centres (53.1%; 47.4% in Belgium vs. 66.7% in the Netherlands, *p* = 0.461). Matrix‐assisted laser desorption/ionisation time‐of‐flight mass spectrometry (MALDI‐TOF‐MS) for identification purposes was available in nearly all centres (30/32 [93.8%]). The possibility of desoxyribonucleic acid (DNA) sequencing for identification was significantly more accessible in the Netherlands 10/12 (83.3%) vs. Belgium (7/19 (36.8%), *p* = 0.024). Beta‐D‐glucan testing was available in 25/32 centres (78.1%), although the majority of these centres outsourced the diagnostic method (20/25 (80%) centres). *Aspergillus* antigen testing was available for 30/32 (93.8%) of centres. In 29/32 (90.6%), of which 10 centres outsourced the analysis, enzyme immunoassay was available. The survey did not differentiate between targeted antigens. *Aspergillus* lateral flow testing was used in 24/32 (75.0%, 11/24 (45.8%) outsourced). Lateral flow testing was implemented more in the Netherlands compared to Belgium (91.7% vs. 68.4%), but this difference was not statistically significant (*p* = 0.202). When available, lateral flow testing was more outsourced in Belgium compared to the Netherlands (*p* = 0.037). *Aspergillus* PCR testing was more frequently available in the Netherlands compared to Belgium (11 centres (91.7%) in the Netherlands versus 12 centres (63.2%) in Belgium), but also here this difference was not significant (*p* = 0.108). Mucorales PCR testing was available in 18/32 centres (56.3%) similar in the Netherlands and Belgium. *Candida* antigen testing was available in 11/32 centres (34.4%), while *Candida* PCR in 18/32 (56.3%). *Histoplasma* antigen testing was available in 17/32 centres (53.1%). In Belgium this was not provided onsite (anymore) but shipped to the Netherlands instead. In the Netherlands *Histoplasma* PCR is performed onsite in two centres (a combined ECMM reference centre). *Histoplasma* serology (immunodiffusion) is performed onsite in one centre in the Netherlands but is not performed in Belgium. PCR for *Pneumocystis jirovecii* was available in all but one centre (96.9%). When available, overall, PCR testing was outsourced significantly more in Belgium compared to the Netherlands (31/49 (63.3%) vs. 7/39 (17.9%), *p* < 0.001).

Antifungal susceptibility testing was available in 29 institutions (90.6%) (16/19 [84.2%] of centres in Belgium and all centres in the Netherlands [*p* = 0.265]). Antifungal susceptibility technology was diverse. In 12 centres (37.5%) broth microdilution using CLSI standards is used and in 15 (46.9%) using EUCAST standards. Twelve centres (37.5%) use gradient MIC strip testing and 13 centres (40.6%) have the VITEK method available.

Availability of therapeutic possibilities is summarised in Table 2. Amphotericin B (all formulations) is available in 26/32 (81.3%) of centres (liposomal amphotericin B in 26 (81.3%), amphotericin B deoxycholate in 11 (34.4%) and amphotericin B lipid complex in nine [28.1%]). In half of the centres, treatment with flucytosine is available while therapeutic drug monitoring (TDM) of flucytosine is available in only 13 centres (40.6%). Triazoles are available in 30/32 centres (93.8%): fluconazole in all 30, isavuconazole in 28, itraconazole in 22, posaconazole in 24 and voriconazole in 28. TDM of posaconazole is available in 24 centres (75.0%) and of voriconazole is possible in 26 centres (81.3%). TDM was outsourced significantly more in Belgium compared to the Netherlands (21.1%–52.6% vs. 8.3%–16.7% (*p* = 0.009)). Considering the echinocandins, anidulafungin was rarely used in Belgium (5.3%) while this is the most frequently used echinocandin in the Netherlands (75.0%) (*p* < 0.001).

**TABLE 2:** Availability of therapeutic possibilities.

	Overall		BE		NL		Comparing BE vs. NL	LU	
	*n*	%	*n*	%	*n*	%	*p*	*n*	%
Available antifungals									
Amphotericin B	27	84.4	15	78.9	11	91.7	0.624	1	100.0
Amphotericin B deoxycholate	11	34.4	4	21.1	7	58.3	*0.056*	0	0.0
Amphotericin B lipid complex	9	28.1	6	31.6	3	25.0	1.000	0	0.0
Amphotericin B liposomal	27	84.4	15	78.9	11	91.7	0.624	1	100.0
Echinocandins	29	90.6	17	89.5	11	91.7	1.000	1	100.0
Anidulafungin	11	34.4	1	5.3	9	75.0	***< 0.001***	1	100.0
Caspofungin	24	75.0	16	84.2	8	66.7	0.384	1	100.0
Micafungin	23	71.9	14	73.7	9	75.0	1.000	0	0.0
Triazoles	30	93.8	18	94.7	11	91.7	1.000	1	100.0
Fluconazole	30	93.8	18	94.7	11	91.7	1.000	1	100.0
Isavuconazole	28	87.5	17	89.5	10	83.3	0.630	1	100.0
Itraconazole	23	71.9	13	68.4	9	75.0	1.000	1	100.0
Posaconazole	25	78.1	14	73.7	10	83.3	0.676	1	100.0
Voriconazole	29	90.6	17	89.5	11	91.7	1.000	1	100.0
Flucytosine	16	50.0	9	47.4	7	58.3	0.716	0	0.0
Terbinafine	22	68.8	12	63.2	10	83.3	0.418	0	0.0
Therapeutic drug monitoring	27	84.4	17	89.5	9	75.0	0.350	1	100.0
Flucytosine	13	40.6	6	31.6	7	58.3	0.262	0	0.0
Onsite	7	21.9	2	10.5	5	41.7		0	0.0
Outsourced	6	18.8	4	21.1	2	16.7		0	0.0
Itraconazole	18	56.3	10	52.6	7	58.3	1.000	1	100.0
Onsite	10	31.3	5	26.3	5	41.7		0	0.0
Outsourced	8	25.0	5	26.3	2	16.7		1	100.0
Posaconazole	25	78.1	16	84.2	8	66.7	1.000	1	100.0
Onsite	12	37.5	6	31.6	6	50.0		0	0.0
Outsourced	13	40.6	10	52.6	2	16.7		1	100.0
Voriconazole	27	84.4	17	89.5	9	75.0	0.384	1	100.0
Onsite	16	50.0	7	36.8	8	66.7		1	100.0
Outsourced	11	34.4	10	52.6	1	8.3		0	0.0

*Note:* *p*‐value below 0.05 in bold/italic, trend to significance in italic.

Abbreviations: BE, Belgium; LU, Luxembourg; NL, Netherlands.

## 4 Discussion

This study provides an overview of the results of a survey regarding access to diagnostic and treatment methods for IFI management, including 32 representative centres from the Benelux. For Belgium, most responders came from the Dutch‐speaking part of the country (Flanders region). Review of the 32 included centres showed a good representation of the clinical mycological field in this region, with reference centres, university centres, and large peripheral centres included. Availability of diagnostics for the most prevalent fungal infections, candidiasis and aspergillosis, in general, was quite good. However, in more than 40% of centres, laboratory procedures were outsourced, making it important to think on logistic elements. This region in Europe is especially of interest as mould antifungal resistance is an emerging problem, primarily as a result of the widespread use of antifungals in agriculture [18, 19, 22, 23, 26, 27].

One of the main concerns that came out of the survey was that microscopy—although available in all of the participating centres receiving patient samples—is not always routinely performed when IFI is suspected, although being principally the golden standard in diagnosing IFI and providing an additional benefit to culture alone [28]. However, the performance of microscopy and the use of optical brighteners (i.e., calcofluor white, blankophor) as a staining dye was still slightly higher compared to countries with the same GDP as found in a pan‐European survey [12]. Expertise is necessary to effectively perform microscopy, but it is vital for effective patient care management. Recent findings show an incidence of mixed mould infections of around 12% which is higher than previously estimated [29, 30, 31], and given the varied treatment approaches necessary by distinct fungal infections and the critical importance of timely diagnosis on patient outcomes, microscopy emerges as a pivotal tool for early identification complemented by molecular methods. Moreover, precise identification of the causative fungus—in contrast to other microbiological tests only suggesting the presence of an IFI—aids in distinguishing among three primary scenarios when a patient with the suspicion of IFI is deteriorating: (1) resistance to antifungal treatment, (2) fungal coinfection, or (3) suboptimal dosing of antifungal drugs.

Although mainly centres of interest were included in the survey, being involved in patient care of immunocompromised patients, many of the diagnostic procedures were outsourced. Outsourcing of certain tests could lead to a delay in diagnosis leading subsequently to a delay in adequate treatment initiation which potentially has an impact on the outcome of the patients. The pan‐fungal marker beta‐D‐glucan for example was—when available—outsourced in 80.0% of cases. And outsourcing, with its inherent delay, might diminish the utility of being a marker for *Pneumocystis jirovecii*, underscoring the further need for single‐sample and more rapid testing [32, 33]. Point of care lateral flow tests, provide the possibility of more rapid and single sample testing, were already more frequently implemented compared to previous reports [12, 34]. As lateral flow tests in this survey were questioned overarching and not product specific, and as performance and available literature of these tests are contingent upon the specific test and manufacturer, this could limit accurate interpretation [34]. Treating physicians must be aware of the (test specific) limitations of these assays, and robust clinical validation is necessary to truly conclude the place and usefulness of these tests in the diagnostic armamentarium. With the Benelux being a high‐income region, we would advocate expert diagnostics instead of compromising for less specific tests, but more rapid tests could be useful to bridge certain limitations linked to logistics (transportation time, testing on weekends and holidays).

Sample transportation may cause a delay in obtaining results, thereby compromising adequate treatment adjustments. On the other hand, being all geographically small, the countries in the Benelux can facilitate inter‐hospital transport and external analyses. In Belgium, most diagnostic tests are consolidated within two specialised centres of expertise (one in the Dutch speaking part of Belgium and one in the French speaking part) [35]. In the Netherlands—although being geographically around 30% larger—diagnostic testing is also consolidated in two specialised centres of expertise with joint accreditation [36].

With an incidence of 7%–13%, *Aspergillus fumigatus* azole‐resistance is particularly high in the Netherlands and Belgium, emphasising the importance of susceptibility testing in IFI [19, 22]. Regarding methods involved in susceptibility testing such as species identification by MALDI‐TOF MS, culture, and PCR, availability was good but could still be improved. The use of MALDI‐TOF MS in Benelux laboratories was widespread with 29/31 centres in Belgium and all centres in the Netherlands and Luxembourg. Belgium and the Netherlands were among the first countries, 2 years after the introduction of *Candida auris* in Europe, to start external quality assessment of clinical microbiological laboratories to identify *Candida auris*. At that time, MALDI‐TOF MS enabled only a correct identification of *Candida auris* in 57.7% of Belgian and 74% of Dutch laboratories [37, 38]. Six years later, the performance approaches 100%, which is of utmost importance for prevention and control of the spread of *Candida auris* [39]. *Aspergillus* PCR, having the additional benefit to look for (genotypic) azole resistance [40], was only available in 63.2% of Belgian centres, which was significantly less than in the Netherlands, although *Aspergillus fumigatus* azole‐resistance is an emerging problem in Belgium. The survey did not specify if in case PCR was available, this also included resistance analysis. The more frequent use of *Aspergillus* PCR in the Netherlands could partially be explained by the fact that national guidelines suggest empiric combination therapy in areas where *Aspergillus fumigatus* azole‐resistance is expected to be more than 10%, and subsequent PCR results should guide escalation to amphotericin B in case of resistance or to maintain with combination therapy if susceptibility results are unavailable [41].

Mucorales PCR is able to early detect invasive mucormycosis days and even weeks before conventional methods (culture, histopathology) [42]. In addition, it is a very sensitive tool to help differentiate a Mucorales and *Aspergillus* coinfection from a case of *Aspergillus fumigatus* azole‐resistance aspergillosis. Therefore, early introduction of the Mucorales PCR, possibly in a context of screening, could impact patient outcome [30, 31, 43, 44]. More recent studies show that more than half of diagnoses of mucormycosis rely solely on PCR, making it plausible these will be implemented in the consensus definitions in the future [45, 46, 47]. However, the possibility of Mucorales PCR testing is only provided in 56.3% of the centres, although most participants registered that they were involved in the treatment of patients at risk.

Part of the deficit in certain diagnostic tests as well as the need for outsourcing in Belgium, in contrast to the Netherlands, could be explained by the fact that many fungal diagnostic tests are not reimbursed by (Rijksinstituut voor Ziekte‐ en Invaliditeitsverzekering)/INAMI (Institut National d'Assurance Maladie‐Invalidité), the national institute for health and disability insurance in Belgium, managing the country's social security system related to healthcare, overseeing the reimbursement of medical expenses, the organisation of healthcare services, and the coordination of various health‐related benefits. If laboratories want to conduct tests which are not reimbursed, they will not receive any financial compensation unless they bill the patients or other laboratories directly. In addition, sometimes a test is only reimbursed when performed in a reference centre. But currently, the costs of the requested tests are significantly exceeding the budget granted to the reference centre. The reimbursement policies for diagnostic tests in Belgium should be re‐evaluated to ensure they remain effective and equitable, for example by granting nomenclature codes that enable invoicing for essential tests. This reassessment is particularly urgent in the context of the European Union (EU) In Vitro Diagnostic Regulation (IVDR), which no longer permits the routine use of Laboratory Developed Tests (LDTs). As a result, several CE/IVD‐labelled fungal molecular diagnostics may be withdrawn from the market, given that upgrading them to meet IVDR compliance is not always economically viable. These regulatory changes will likely impact diagnostic availability across the Benelux and EU, and national policies must adapt to mitigate potential access gaps.

Concerning antifungal treatment availability, it is noteworthy that amphotericin B was not accessible in all centres—liposomal amphotericin B was available in only 84.4% of them. This is particularly striking given that both Dutch and European guidelines recommend initiating empiric treatment with either a combination of an azole and an echinocandin or with amphotericin B in regions where local azole resistance rates exceed 10%, as is the case in certain areas of Belgium and the Netherlands [5, 36]. *Aspergillus fumigatus* azole‐resistance rates in Luxembourg are lacking, with isavuconazole as the only mould‐active treatment available in the country's participating centre. Second, 12.6% of all centres reported a moderate or high incidence of mucormycosis for which amphotericin B remains the first‐line treatment [7]. And third, also in certain conditions in which azoles are considered unsafe or are not sufficiently studied (pregnancy, paediatric cases) amphotericin B availability is crucial [5, 48]. Another notable observation regarding the treatment was that when selecting an echinocandin, anidulafungin was most often the first choice in the Netherlands, while this was the opposite in Belgium. Advantages of anidulafungin compared to caspofungin can be discussed, with evidence available that clinicians sometimes prefer the use of anidulafungin treatment in sicker patients with worse prognosis and comorbidities as renal and hepatic profile is safe [49]. In addition to diagnostic tests, TDM is also more often outsourced in Belgium.

A limitation of the study is that the availability of metagenomic next‐generation sequencing (mNGS) and whole genome sequencing (WGS) was not included in the study (only DNA sequencing for species identification). As mNGS is a promising approach for hypothesis‐free diagnosis of IFI, with its main drawback being the expertise and costs associated with this method, it would have been interesting to evaluate this as well [50, 51]. It would also be of interest to know if new point‐of‐care tests or lateral flow assays for rare mould infections are implemented, for example, in a study context. Now only routine clinical tests were questioned, but as new tests for rare difficult‐to‐treat infections are under development, maybe these are already under evaluation somewhere [52, 53]. Potential bias may exist considering that respondents to the questionnaire may predominantly represent mycology‐focused laboratories. Furthermore, the survey did not inquire about the availability of radiological procedures, despite the pivotal role of imaging in diagnosing invasive fungal infections [45, 54]. With positron emission tomography—computed tomography (PET‐CT) expected to assume greater significance in future IFI diagnostics, insights into its availability in specific centres would have been valuable [55, 56, 57]. However, given the advanced and very well accessible healthcare infrastructure across all three countries [58, 59], it is unlikely that radiology poses a significant constraint on achieving accurate diagnoses. A last limitation is the inclusion of only one centre from Luxembourg, resulting in its exclusion from the statistical comparison. However, given Luxembourg's status as a small country with only one academic centre, this inclusion may offer a representative perspective, and as Luxembourg was lacking in the large European survey, this remains an additional asset.

The Benelux mostly has excellent access to diagnostic tools, antifungal treatment, and TDM. With the countries being geographically small, centralising in reference centres improves expert diagnostics. There seems to be a larger availability of antifungal susceptibility test technologies in Belgium and the Netherlands compared to Europe in general. However, based on this survey, some points for improvement could be highlighted. Microscopy should be standard‐of‐care every time an IFI is suspected, but this is not yet the case. Also, the implementation of molecular testing and other tests focusing on antifungal susceptibility and identification of coinfections in these areas of high *Aspergillus fumigatus* azole‐resistance could be improved by reworking reimbursement criteria.

## Author Contributions

**Robina Aerts:** conceptualization, methodology, writing – review and editing, writing – original draft, investigation, validation, project administration, formal analysis, supervision. **Lize Cuypers:** conceptualization, funding acquisition, writing – original draft, methodology, validation, writing – review and editing, project administration, supervision. **Eelco F. J. Meijer:** conceptualization, funding acquisition, writing – original draft, methodology, validation, writing – review and editing, project administration, supervision. **Michel Kohnen:** conceptualization, funding acquisition, writing – original draft, methodology, visualization, writing – review and editing, project administration, supervision. **Jacques F. Meis:** funding acquisition, writing – original draft, conceptualization, methodology, visualization, writing – review and editing, project administration, supervision. **Oliver A. Cornely:** conceptualization, investigation, funding acquisition, writing – original draft, methodology, validation, writing – review and editing, project administration, software, supervision, resources, data curation. **Katrien Lagrou:** conceptualization, funding acquisition, writing – original draft, methodology, visualization, writing – review and editing, project administration, supervision. **Jon Salmanton‐García:** conceptualization, investigation, funding acquisition, writing – original draft, methodology, validation, visualization, writing – review and editing, software, formal analysis, project administration, resources, data curation, supervision.

## Conflicts of Interest

R.A. received PhD funding from the Flemish Cancer Society (Kom op Tegen Kanker) and a research grant and travel support from Pfizer and Gilead Sciences, outside of the submitted work. L.C. declares no conflicts of interest. E.F.J.M. received research grants from Mundipharma and Scynecis, is on the scientific advisory board for Pfizer and has received speaker fees from Gilead Sciences, outside of the submitted work. M.K. declares no conflicts of interest. J.F.M. declares no conflicts of interest. O.A.C. reports grants or contracts from iMi, iHi, DFG, BMBF, Cidara, DZIF, EU‐DG RTD, F2G, Gilead, MedPace, MSD, Mundipharma, Octapharma, Pfizer, Scynexis; consulting fees from Abbvie, AiCuris, Basilea, Biocon, Boston Strategic Partners, Cidara, Elion, Gilead, GSK, IQVIA, Janssen, Matinas, MedPace, Menarini, Melinta, Molecular Partners, MSG‐ERC, Mundipharma, Noxxon, Octapharma, Pardes, Partner Therapeutics, Pfizer, PSI, Scynexis, Seres, Seqirus, Shionogi, Prime Meridian Group; speaker and lecture honoraria from Abbott, Abbvie, Al‐Jazeera Pharmaceuticals/Hikma, amedes, AstraZeneca, Gilead, GSK, Grupo Biotoscana/United Medical/Knight, Ipsen Pharma, Medscape/WebMD, MedUpdate, MSD, Moderna, Mundipharma, Noscendo, Paul‐Martini‐Stiftung, Pfizer, Sandoz, Seqirus, Shionogi, streamedup!, Touch Independent, Vitis; participation on a DRC, DSMB, DMC, or Advisory Board for AstraZeneca, Cidara, IQVIA, Janssen, MedPace, Melinta, PSI, Pulmocide, Vedanta Biosciences, outside of the submitted work. K.L. received consultancy fees from Mundipharma, speaker fees from Pfizer, Gilead, Mundipharma, and FUJIFILM Wako chemicals Europe GmbH, a service fee from TECOmedical, a fee for Advisory Board participation from Pfizer, and travel support from Pfizer, Gilead, and AstraZeneca, outside of the submitted work. J.S.G. has received speaker honoraria from AstraZeneca, Gilead, Pfizer, and Menarini, and has been a member of an advisory board for Pfizer, outside of the submitted work.

## Supporting information


Data S1.

## Data Availability

The corresponding author can provide the data supporting the findings of this study upon a reasonable request.
