# Supplementary material for: ESCMID‐EFISG Survey on Diagnostic and Therapeutic Capacity for Invasive Fungal Infections in Belgium, the Netherlands, and Luxembourg: A Focus on High Azole Resistance
Source: Mycoses. 2025 Jul 12;68(7):e70092. doi: 10.1111/myc.70092 (PMC12254878; doi:10.1111/myc.70092)
Supplement: Supplementary file 1 — Data S1. [file MYC-68-e70092-s001.docx]

**SUPPLEMENTARY MATERIAL**

**Appendix 1.** Summary of the survey.

1. ***Institution Profile***
   1. Your position
      1. Attending Physician
      2. Attending Physician - Infectious Diseases Specialist
      3. Clinical microbiologist
      4. Director
      5. Infection Control Practitioner
      6. Laboratory Professional
      7. Professor
      8. Other
   2. Contact information
      1. Your name
      2. Your e-mail address
   3. Institution
      1. Institute
      2. Department
   4. Location of your institution
      1. City
      2. Region/State
      3. Country
   5. Institution profile
      1. Day-Hospital
      2. Dialysis Clinic
      3. Federal Institute / Research Hospital
      4. Oncology Clinic
      5. Private Hospital
      6. Private Laboratory
      7. Public Hospital
      8. University Hospital
      9. Other. Please, specify:
   6. Institution size - number of beds
      1. Overall
      2. Adult intensive care beds
      3. Paediatric/Neonatal intensive care beds
   7. Does your institution take care of patients with any of the following conditions? *Please answer each question with yes, no or unknown.*
      1. COVID-19
      2. Diabetes mellitus
      3. Hematology
      4. HIV/AIDS
      5. Neonatal Intensive Care Unit
      6. Oncology
      7. Parenteral nutrition
      8. Solid organ transplantation
      9. Stem cell transplantation
   8. Does your institution have a microbiology laboratory?
      1. Yes, in place
      2. Yes, outsourcing laboratory services
      3. No
   9. Where is diagnostic mycological procedure performed?
      1. Always in our institution
      2. Part in our institution / part outsourced
      3. Totally outsourced
      4. We do not have access to mycological diagnostic tools
2. ***Perceptions on invasive fungal disease in your institution***
   1. Please rate the incidence of invasive fungal infections in your institution from very low (1) to very high (5)
   2. Please rate the incidence of mucormycosis in your institution from very low (1) to very high (5)
   3. Pathogens of highest importance
      1. *Aspergillus* spp.
      2. *Candida* spp.
      3. *Cryptococcus* spp.
      4. *Fusarium* spp.
      5. *Histoplasma* spp.
      6. Mucorales
   4. What is the approximate number of samples (per month) processed in your mycology laboratory?
      1. TOTAL number of samples
      2. BLOOD samples
      3. BAL (bronchoalveolar lavage) samples
      4. TISSUE (from biopsies) samples
      5. URINE samples
   5. Please indicate all available drugs for antifungal treatment in your institution. *Please answer each question with yes, no or unknown.*
      1. Amphotericin B deoxycholate
      2. Amphotericin B lipid complex
      3. Amphotericin B liposomal
      4. Amphotericin B - other formulations
      5. Anidulafungin
      6. Caspofungin
      7. Fluconazole
      8. Flucytosine (5-FC)
      9. Isavuconazole
      10. Itraconazole
      11. Micafungin
      12. Posaconazole
      13. Terbinafine
      14. Voriconazole
3. ***Microscopy***
   1. Which methodologies are used in fungal microscopy? *Please answer each question with yes, no or unknown.*
      1. Calcofluor white
      2. Giemsa stain
      3. China/India ink
      4. Potassium hydroxide
      5. Silver stain
      6. Others
   2. How frequently is microscopy performed when a fungal disease is suspected from never (1) to always (5)? (e.g., in sterile clinical samples or BAL)
   3. Do you have access to fluorescence dyes?
   4. When cryptococcosis is suspected is direct examination in body fluids available?
      1. Yes, India ink
      2. Yes, other dyes
      3. No
   5. When pneumocystis is suspected is silver stain performed?
      1. Yes
      2. No
   6. When mucormycosis is suspected, is direct microscopy with optical brighteners performed?
      1. Yes
      2. No
4. ***Culture and Fungal Identification***
   1. Are automated blood cultures available in case of fungemia suspicion?
      1. Yes
      2. No
   2. Please mark all methods used for fungal cultures. *Please answer each question with yes, no or unknown.*
      1. Agar Niger
      2. Chromogen
      3. Lactrimel Agar
      4. Potato Dextrose Agar
      5. Sabouraud
      6. Sabouraud + Chloramphenicol
      7. Sabouraud + Gentamicin
      8. Selective agar (Chloramphenicol + Cycloheximide)
      9. Others
   3. Please select all available test for species identification. *Please answer each question with yes, no or unknown.*
      1. Automated identification (i.e., VITEK^®^, other commercial tests)
      2. Biochemical tests (classic mycology)
      3. DNA sequencing
      4. MALDI – TOF – MS
      5. Mounting medium
   4. Do you have access to antifungal susceptibility tests?
      1. For yeasts
      2. For moulds
      3. For both
      4. None
   5. Which of the following technologies for susceptibility testing are available? *Please answer each question with yes, no or unknown.*
      1. Broth microdilution, using CLSI standards
      2. Broth microdilution, using EUCAST standards
      3. E-test^®^
      4. VITEK^®^
   6. Please choose the answer that best matches the maximum identification capability (of yeasts) in your laboratory
      1. Genus
      2. Genus / species
      3. Genus / species / complex
      4. Genus / species / complex / cryptic species
   7. Please choose the answer that best matches the maximum identification capability (of moulds) in your laboratory
      1. Genus
      2. Genus / species
5. ***Serology***
   1. Which of the following serology tests (antibody detection) are available? *Please answer each question with yes, only at an outsourced laboratory, no or unknown.*
      1. *Aspergillus* spp.
      2. *Candida* spp.
      3. *Histoplasma* spp.
      4. *Paracoccidioides* spp.
6. ***Antigen Detection***
   1. Which of the following antigen detection tests are available? *Please answer each question with yes, only at an outsourced laboratory, no or unknown.*
      1. *Aspergillus* (lateral flow device)
      2. *Aspergillus* galactomannan (immunoenzymatic sandwich microplate assay)
      3. *Aspergillus* galactomannan (lateral flow assay)
      4. *Candida* antigen
      5. *Cryptococcus* (lateral flow assay)
      6. *Cryptococcus* (latex agglutination test)
      7. *Histoplasma*
      8. Beta-d-glucan
7. ***Molecular Tests***
   1. Which of the following molecular tests are available? *Please answer each question with yes, only at an outsourced laboratory, no or unknown.*
      1. *Aspergillus* PCR
      2. *Candida* PCR
      3. *Pneumocystis* PCR
      4. Mucorales PCR
      5. PCR for other fungi
      6. Other molecular tests

1. ***Therapeutic Drug Monitoring (TDM)***
   1. Does your institution have access to therapeutic drug monitoring of antifungal agents? *Please answer each question with yes, only at an outsourced laboratory, no or unknown.*
      1. 5-flucytosine
      2. Itraconazole
      3. Posaconazole
      4. Voriconazole
